# P-181. Limited oxygen therapy capacity against COVID-19 and the public health response in Zambia 2021

**DOI:** 10.1093/ofid/ofae631.386

**Published:** 2025-01-29

**Authors:** Suilanji Sivile, Takeaki Imamura, Tadatsugu Imamura, Paul Msanzya Zulu, Masuzyo Zyambo, Papytcho Ntambwe, Ndaba Spuka, Julien Chomba, Anita Enane, O T IP O Sikanga, Alphonsina Hamalala, Hamuganyu Innocent, Christopher Chanda, Lloyd Mulenga

**Affiliations:** Zambia Ministry of Health, Lusaka, Lusaka, Zambia; Tohoku University, Sendai, Miyagi, Japan; Japan International Cooperation Agency, Lusaka, Lusaka, Zambia; University Teaching Hospital, Lusaka, Lusaka, Zambia; Levy Mwanawasa Hospital, Lusaka, Lusaka, Zambia; Livingstone Central Hospital, Livingstone, Southern, Zambia; Maina Soko Hospital, Lusaka, Lusaka, Zambia; Arthur Davison Children’s Hospital, Ndola, Copperbelt, Zambia; World Health Organization Zambia Country Office, Lusaka, Lusaka, Zambia; World Health Organization Zambia Country Office, Lusaka, Lusaka, Zambia; Ministry of Health, Zambia, Environmental Health, Lusaka, Lusaka, Zambia; Ministry of Health, Zambia, Environmental Health, Lusaka, Lusaka, Zambia; University Teaching Hospital, Lusaka, Lusaka, Zambia

## Abstract

**Background:**

The coronavirus disease 2019 (COVID-19) pandemic revealed the limited capacity of intensive oxygen therapy in Zambia, especially in provincial medical facilities.

Zambia COVID-19 treatment centers with newly installed HFNC and NPPV devices in 2021

**Figure:**
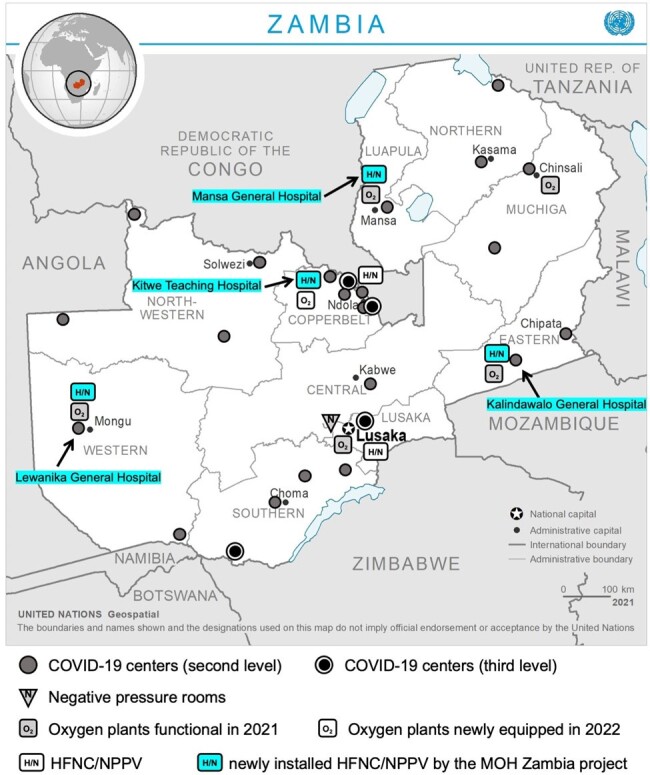

**Methods:**

The Ministry of Health (MoH) conducted a clinical chart review of COVID-19 cases admitted to 11 COVID-19 treatment centers across Zambia to evaluate oxygen therapy practices and clinical outcomes. Then, MoH launched a multidisciplinary capacity-building project targeting four national COVID-19 treatment centers in the provinces aiming to deploy high-flow nasal cannula (HFNC) and non-invasive positive pressure ventilation (NPPV) coupled with training for healthcare workers. The chart review and the reporting of the project were approved by National Health Research Authority, Zambia (NHRA000006/15/10/2021, NHRA00003/25/04/2022), and Tohoku University, Japan (2021-1-983, 2022-1-287).

**Results:**

Among 1,687 cases of severe/critical COVID-19, 84 cases (5%) were treated without oxygen, 687 (41%) were administered with oxygen only via nasal cannula, 759 (45%) via conventional masks, and 157 cases (9%) received intensive oxygen therapy, including HFNC, NPPV, and invasive mechanical ventilation. Compared to the capital Lusaka, COVID-19 cases treated in the provinces were more likely to receive oxygen administration only via nasal cannula and less likely to receive intensive oxygen therapy. To strengthen the intensive oxygen therapy capacity in the provinces, MoH installed HFNC and NPPV devices in four provincial medical facilities equipped with oxygen plants and piping in October and November 2021. MoH also provided a five-day training program for healthcare workers focusing on clinical knowledge/skills of HFNC and NPPV in COVID-19 and infection prevention and control in aerosol-generating procedures.

**Conclusion:**

Our study identified the inequity of intensive oxygen therapy capacity between Lusaka and the provinces in Zambia and the need for further strengthening. A multidisciplinary approach coordinating national experts was practical in rapidly deploying HFNC and NPPV amid the COVID-19 pandemic.

**Disclosures:**

**All Authors**: No reported disclosures

